# Supplementary material for: Boosting Expression of a Specifically Targeted Antimicrobial Peptide K in Pichia pastoris by Employing a 2A Self-Cleaving Peptide-Based Expression System
Source: Antibiotics (Basel). 2024 Oct 18;13(10):986. doi: 10.3390/antibiotics13100986 (PMC11505851; doi:10.3390/antibiotics13100986)
Supplement: Supplementary file 1 [file antibiotics-13-00986-s001.zip › antibiotics-3231475-supplementary.pdf]

Supporting Information

Boosting expression of a selective targeted antimicrobial peptide K in *Pichia pastoris* by employing a 2A self-cleaving peptide-based expression system and optimizing the fermentation conditions.

ZHU Yunhui, LI Yuwen, FANG Yuxin, HU Mingyang, ZHAO Lu, SUI Mingrui, DONG Na*

Laboratory of Molecular Nutrition and Immunity, College of Animal Science and Technology, Northeast Agricultural University, Harbin, PR China.

* Corresponding authors

Prof. Na Dong, E-mail address: ndong@neau.edu.cn; Tel.: +86 0451 55190722;

**Item**	**Title**
**Figure. S1.**	Screening of positive recombinant GS115 on MM plates.
**Figure. S2.**	Blast between gene sequencing and gene synthesis for transformants.
**Figure. S3**	WB for recombinant peptide in supernatant.
**Table. S1.**	The nucleic acid sequence of inserted fragment.

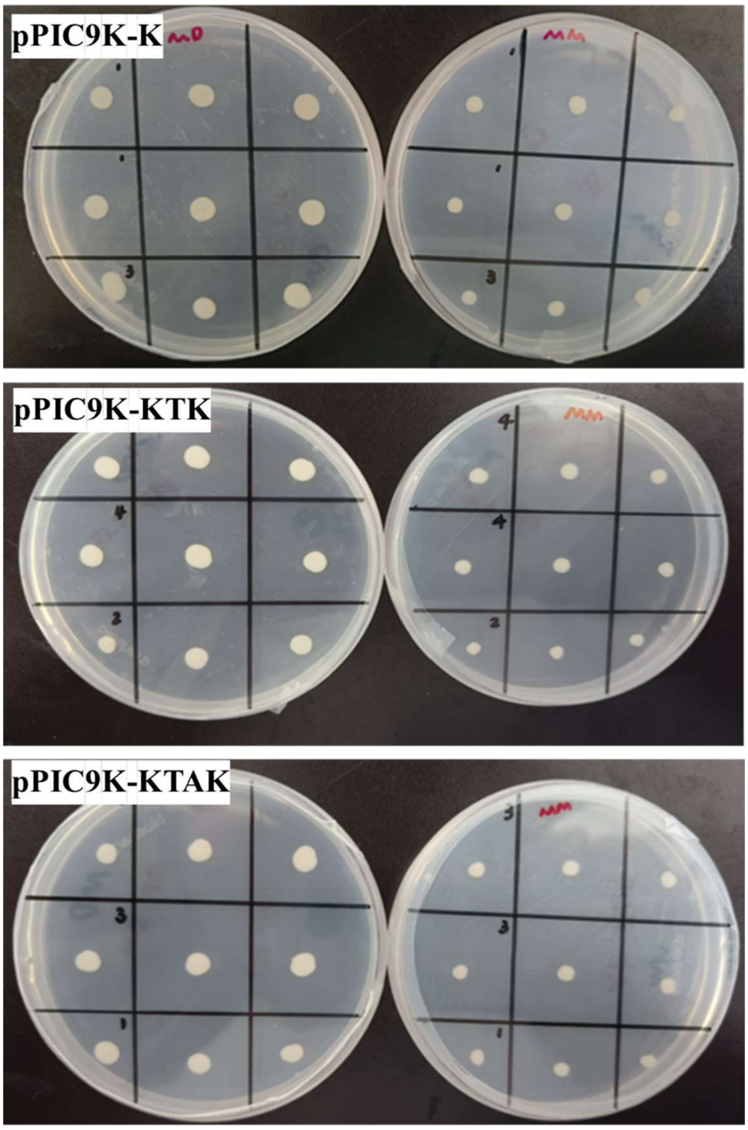

Figure. S1. Screening of positive recombinant GS115 on MM plates.

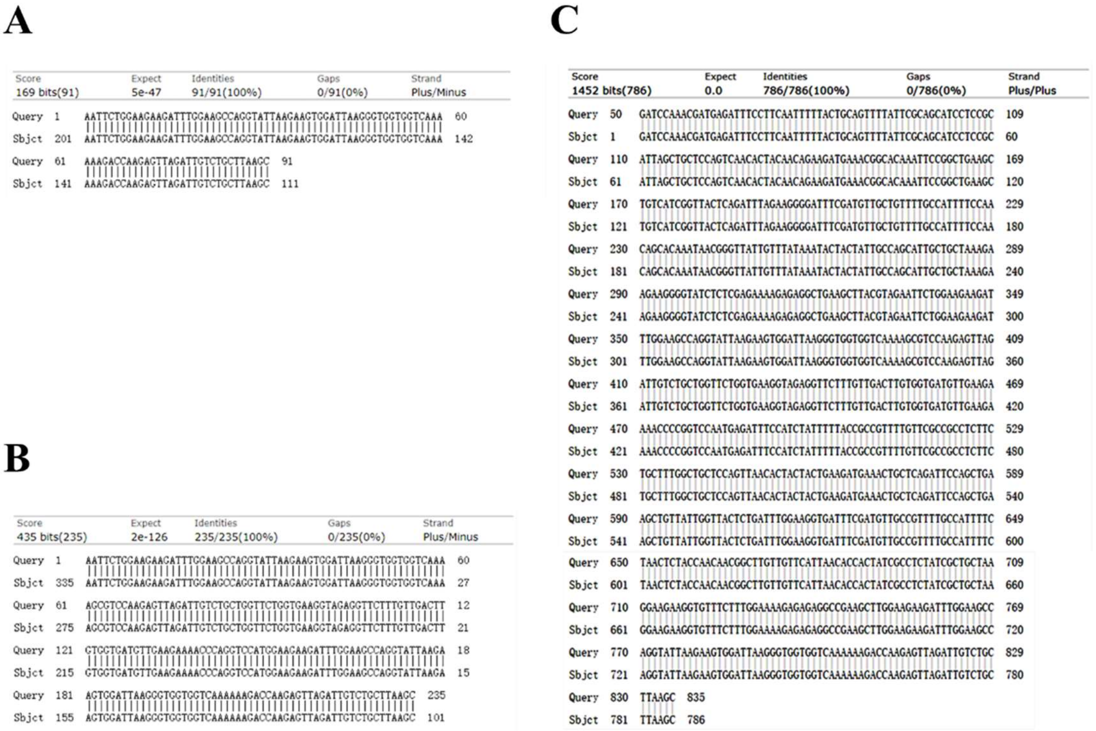

Figure. S2. Blast between gene sequencing and gene synthesis for transformants. **A**. pPIC9K-K; **B.** pPIC9K-KTK, **C**. pPIC9K-KTAK.

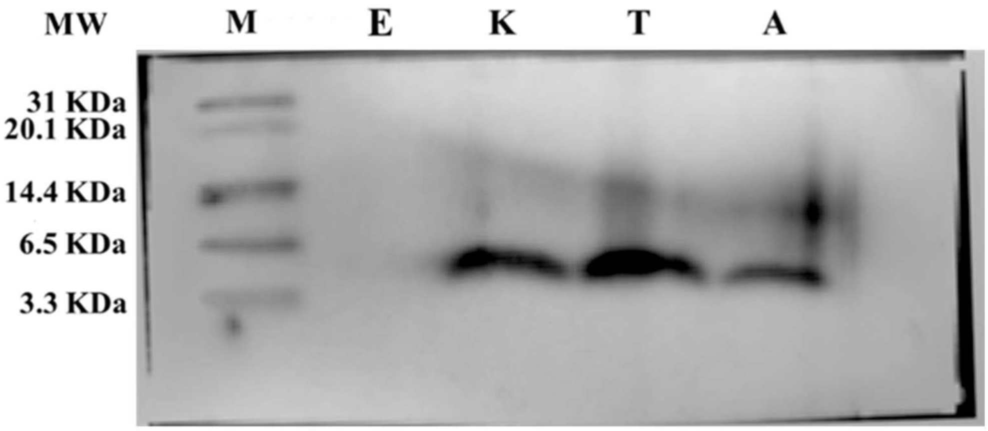

Figure. S3. WB for recombinant peptide. M: marker; E: empty vector; T: KTK; A: KTAK

Table S1. The nucleic acid sequence of inserted fragment.

Items	Nucleic acid sequence
K	TGGAAGAAGATTTGGAAGCCAGGTATTAAGAAGTGGATTAAGG<br>GTGGTGGTCAAAAAAGACCAAGAGTTAGATTGTCTGCT
KTK	TGGAAGAAGATTTGGAAGCCAGGTATTAAGAAGTGGATTAAGG<br>GTGGTGGTCAAAAGCGTCCAAGAGTTAGATTGTCTGCTGGTTCT<br>GGTGAAGGTAGAGGTTCTTTGTTGACTTGTGGTGATGTTGAAGA<br>AAACCCAGGTCCATGGAAGAAGATTTGGAAGCCAGGTATTAAG<br>AAGTGGATTAAGGGTGGTGGTCAAAAAAGACCAAGAGTTAGAT<br>TGTCTGCT
KTAK	GGAAGAAGATTTGGAAGCCAGGTATTAAGAAGTGGATTAAGGG<br>TGGTGGTCAAAAGCGTCCAAGAGTTAGATTGTCTGCTGGTTCTG<br>GTGAAGGTAGAGGTTCTTTGTTGACTTGTGGTGATGTTGAAGAA<br>AACCCCGGTCCAATGAGATTTCCATCTATTTTTACCGCCGTTTTG<br>TTCGCCGCCTCTTCTGCTTTGGCTGCTCCAGTTAACACTACTACT<br>GAAGATGAAACTGCTCAGATTCCAGCTGAAGCTGTTATTGGTTA<br>CTCTGATTTGGAAGGTGATTTCGATGTTGCCGTTTTGCCATTTTC<br>TAACTCTACCAACAACGGCTTGTTGTTCATTAACACCACTATCG<br>CCTCTATCGCTGCTAAGGAAGAAGGTGTTTCTTTGGAAAAGAGA<br>GAGGCCGAAGCTTGGAAGAAGATTTGGAAGCCAGGTATTAAGA<br>AGTGGATTAAGGGTGGTGGTCAAAAAAGACCAAGAGTTAGATT<br>GTCTGCTT
